# SARS coronavirus 2: from genome to infectome

**DOI:** 10.1186/s12931-020-01581-z

**Published:** 2020-12-01

**Authors:** Meghana Rastogi, Neha Pandey, Astha Shukla, Sunit K. Singh

**Affiliations:** Molecular Biology Unit, Institute of Medical Sciences, Banaras Hindu University, Varanasi, 221005 India

**Keywords:** Coronaviruses, COVID-19, SARS-CoV-2, Spike glycoprotein, ACE2 receptors, Acute respiratory distress syndrome (ARDS)

## Abstract

Severe acute respiratory syndrome coronavirus-2 (SARS-CoV-2) belongs to the group of Betacoronaviruses. The SARS-CoV-2 is closely related to SARS-CoV-1 and probably originated either from bats or pangolins. SARS-CoV-2 is an etiological agent of COVID-19, causing mild to severe respiratory disease which escalates to acute respiratory distress syndrome (ARDS) or multi-organ failure. The virus was first reported from the animal market in Hunan, Hubei province of China in the month of December, 2019, and was rapidly transmitted from animal to human and human-to-human. The human-to-human transmission can occur directly or via droplets generated during coughing and sneezing. Globally, around 53.9 million cases of COVID-19 have been registered with 1.31 million confirmed deaths. The people > 60 years, persons suffering from comorbid conditions and immunocompromised individuals are more susceptible to COVID-19 infection. The virus primarily targets the upper and the lower respiratory tract and quickly disseminates to other organs. SARS-CoV-2 dysregulates immune signaling pathways which generate cytokine storm and leads to the acute respiratory distress syndrome and other multisystemic disorders.

## Background

Coronaviruses (CoVs) were first isolated from humans in 1962 [1]. The CoVs were thought to cause only mild respiratory and gastrointestinal infections in human and animals [2]. The outbreaks of Severe acute respiratory syndrome coronavirus 1 (SARS-CoV-1) in 2002–2003 in Guangdong province, China [3] and the Middle East respiratory syndrome coronavirus (MERS-CoV) in the Middle Eastern countries, particularly Saudi Arabia in 2012 [4], changed the prevailing concept on coronavirus infections. Both the viruses originated in bats and their chain of transmission established between animal to human and human to human [5, 6]. A similar outbreak of pneumonia like respiratory infections, reported from the Wuhan city, Hubei province, China in late December 2019 made a new addition to the list of human CoVs. The International Committee on Taxonomy of Viruses (ICTV) has named the novel Coronavirus as Severe acute respiratory syndrome coronavirus 2 (SARS-CoV-2) and the disease caused by this virus has been officially named as COVID-19 by WHO [7]. The COVID-19 has been regarded as Public Health Emergency of International Concern (PHEIC) on January 30, 2020 by WHO [8]. SARS-CoV-2 shares 96% genome similarity with a bat Coronavirus [9–11]. The primary targets are the type-II alveolar epithelial cells and airway-epithelial cells, which highly express the Angiotensin converting enzyme-2 (ACE2) receptor on their surface. The ACE2 receptor is used for internalization, similar to SARS-CoV-1 and Human Coronavirus-229E (HCoV-229E) [12]. The SARS-CoV-2 quickly replicates inside the cells and kick-start the plethora of signaling cascade, from activating the pro-inflammatory response to antiviral response leading to cytokine storm. The virus rapidly disseminates through peripheral blood to other organs like, heart, kidney, liver, spleen, etc. [12, 13]. However, the pathogenicity of SARS-CoV-2 is notably less than SARS-CoV-1 and MERS-CoV, but its high transmissibility led to the pandemic, which resulted in the global lock-down and affected the global health scenario adversely [14]. The rapid development of the diagnostic tools and therapeutics in the form of antivirals and vaccines are the need of an hour to overcome the present situation.

## Epidemiology

SARS-CoV-2 has been identified as a third zoonotic-human coronavirus [15]. The bats are the natural host for SARS-CoV-2 while the intermediate reservoir is still under debate [16–18]. As per the global scenario, about 53.9 million people have been reported to be positive for COVID-19 with 1.31 million confirmed deaths and 34.7 million recovered till November 14, 2020 [19]. The top five worst COVID-19 affected countries include, United States, India, Brazil, France and Russia with > 1.5 million cases till November 14, 2020 [19].

The early reports from USA, China and Italy indicated the SARS-CoV-2 infection among people > 60 years of age [20–22]. However, the recent reports (June–August, 2020) indicated the increased rate of infection (4.5% to 15%) among age groups of 15–29 years [23]. This dramatic shift in the infection cases in terms of age groups may be due to the reversion of younger population to their work place, Universities, colleges and schools etc. [23]. Therefore, the COVID-19-related mortality has a varying age distribution starting from 10 to 80 years of age with a greater number of cases reported among patients suffering from other co-morbid conditions [24]. The case fatality rate (CFR) is defined as total number of deaths to the total number of cases reported. In case of COVID-19 the CFR differs from country to country due to the differences in the medical and health infrastructure, co-morbidities and population age.

The major co-morbid conditions leading to the severity of COVID-19 include 10.5% for cardiovascular disease (CVD), 37.3% for diabetes, 8.3% for chronic obstructive pulmonary diseases (COPD), and 55.4% for hypertension, and 8.1% for cancer patients [25–27].

The sex-disaggregated COVID-19 data, collected from 26 countries indicate that males and females are almost equally susceptible to SARS-CoV-2 infection, however the mortality rate is 2.4 times higher in males compared to females [14, 28, 29]. The high mortality rates among males may be correlated to the co-morbidities like, diabetes, hypertension, cardiovascular diseases, and chronic kidney diseases etc. [30]. The higher levels of circulating ACE2 have been reported in the plasma of males suffering from COVID-than females. This condition indicates the higher levels of ACE2 receptor expression on tissues, which help in virus internalization [31]. Overall, the COVID19 related mortalities have been reported to be higher in males than females due to the differences in immunological, genetic, endocrinological, social and behavioral factors [32].

**Fig. 1:**
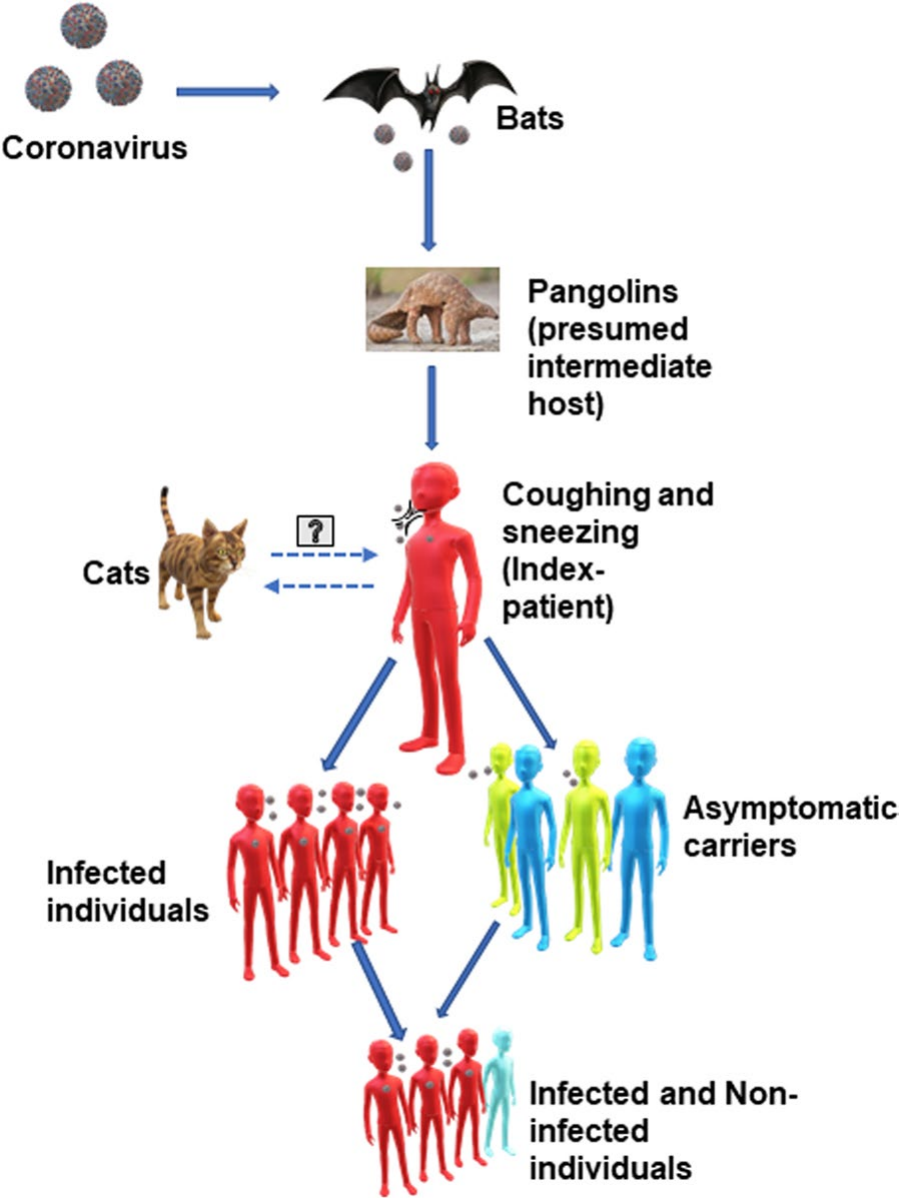
The transmission cycle of SARS-CoV-2. The SARS-CoV-2 originated from bats and pangolins are presumed to be their intermediate amplifying hosts. The virus transmits from animal-to-human to human-to-human. The infected person transmits the virus through cough and sneeze. In the population, there are asymptomatic carrier which spread the virus without any signs or symptoms

The SARS-COV-2 transmission was most probably due to cross-species jump from animal to humans, which first started from wet animal market in Wuhan province, China [33, 34]. The person-to-person transmission established from the visitors who visited Huanan animal market [35]*.* Therefore, the mode of transmission is either through direct human contact, or through the droplets generated during sneezing and coughing of an infected individual (Fig. 1) [36]. The presence of viral RNA in stool samples suggest another route of transmission but so far it has not established very well due to the absence of the live virus in stool samples [37]. The vertical transmission of virus was reported during SARS-CoV-1 and MERS-CoV outbreaks, while the same could not be established so far in the case of SARS-COV-2 [36, 38, 39]. The testing of symptomatic and asymptomatic patients, containment procedures and other precautionary measures like, wearing masks in public places, maintain social distancing, regular use of handwash and hand sanitizers should be adopted to break the chain of transmission of SARS-CoV2 [40].

## Taxonomic status and structure of SARS-CoV-2

Coronaviruses (CoVs) belong to *Coronaviridae* family (subfamily *Coronavirinae*)*,* order *Nidovirales *[10]. The subfamily *Coronavirinae* has been divided into four genera: -*Alphacoronavirus, Betacoronavirus, Gammacoronavirus* and *Deltacoronavirus* [10, 41]. The *Alphacoronavirus* and *Betacoronavirus* are known to infect humans [2]. Bats serve as the evolutionary hosts for the *Alphacoronavirus* and *Betacoronavirus* [42]*.* The whole genome sequencing and phylogenetic analysis classified SARS-CoV-2 as *Betacoronavirus* from the sub-genus *Sarbecovirus*, which also includes SARS-CoV-1 [9, 43]. The mutations, recombination and re-assortments routinely occur in the RNA viruses as a part of the evolutionary process for increasing the genetic diversity. The *Betacoronavirus * have been reported to undergo recombination within bats. The SARS-CoV-2 belongs to Sarbecovirus and shares similarity with two bat derived Coronavirus strains bat-SL-CoVZC45 and bat-SL-CoVZXC21 [43]. The SARS-CoV-2 genome shows 96% similarity to horse-shoe bat virus RaTG13 *Rhinolophus affinis* [9, 44]. The ecological separation of bats from human population makes an obvious note on the presence of an intermediate host, where SARS-CoV-2 develops adaptive changes, before transmitting to humans. This is supported by the difference in the key genomic features of SARS-CoV-2 from RaTG13 and SARS-CoV-1 [45]. Although RaTG13 is 96% similar to SARS-CoV-2, but the receptor binding domain (RBD) of SARS-CoV2 spike protein shares only 85% similarity with the RaTG13 and only one out of six critical amino acid residues of RBD is similar in RaTG13 and SARS-CoV-2 [46–48]. The five of the six amino acid residues differ between the SARS-CoV-1 and SARS-CoV-2 [48]. The SARS-CoV-2 spike proteins contain a polybasic furin cleavage site insertion (residues PRRA) at the junction of S1 and S2, which is probably enhancing the infectivity of the SARS-CoV-2 and is not present in any other Coronavirus [46–48]. The coronaviruses reported in Pangolin exhibit a strong similarity to SARS-CoV-2. The Malayan pangolins *Manis javanica* illegally imported into southern China (Guangdong and Guangxi provinces) were reported to be infected with SARS-CoV-2 related virus [44, 49]. Several SARS-CoV-2 related viruses have been reported in Malayan Pangolins. The sequencing data from these strains show them to be very closely related to SARS-CoV-2 and share 92.4–99.8% sequence identity. The Receptor Binding Motif (RBM) of Spike protein of these strains is also identical to SARS-CoV-2 and differs only in one out of five critical amino acid residues [49–51]. Therefore, SARS-CoV-2 might be a recombinant form of bat and pangolin coronaviruses, and the homologous recombination events might have occurred in spike glycoprotein genes between bat and pangolin CoVs [11, 45]. It has been reported that the cats and ferrets can also get infected with SARS-COV-2 and are susceptible to air-borne transmission.

However, the virus replicates poorly in dogs, pigs and chicken [52–54]. Though, to ascertain the exact pattern and genomic ancestors of the recombination events, a wider sampling of the viral diversity is required to resolve the evolutionary events.

## Genomic organization of SARS-CoV2

**Fig. 2:**
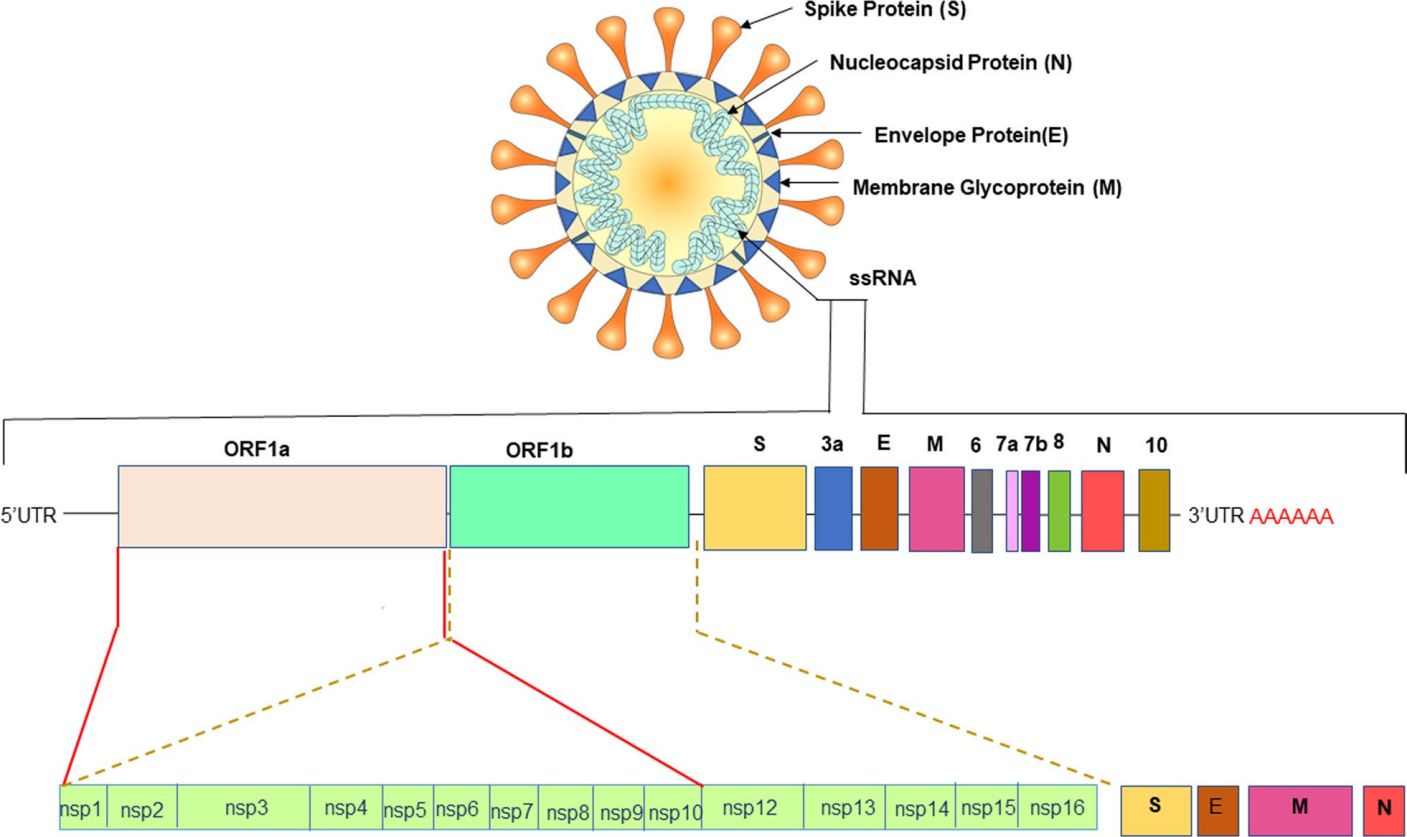
Schematic presentation of the SARS-CoV-2 genome Structure. SARS-CoV-2 has a spherical structure. The virus has an outer lipid envelope, covered with spike glycoprotein. The SARS-CoV-2 represents a typical Betacoronavirus genome organisation. The full-length RNA genome comprises of approximately 29,903 nucleotides and has a replicase complex (comprised of ORF1a and ORF1b) at the 5′UTR. The ORF1a encodes for nsp1–nsp10, while ORF1b encodes for nsp1–nsp16. Four genes that encode for the Structural proteins: Spike gene, Envelope gene, Membrane gene, Nucleocapsid gene and a poly (A) tail at the 3′UTR. The accessory genes are distributed in between the structural genes

The SARS-CoV-2 is a single stranded positive RNA virus of ~ 29.9 kB in size. The SARS-CoV-2 has 14 open reading frames (ORFs), which encodes for 27 different proteins [55]. It has 5′ untranslated region (UTR), replication complex (ORF1a and ORF1b), Spike (S) gene, Envelope (E) gene, Membrane (M) gene, Nucleocapsid (N) gene, 3′ UTR, several unidentified non-structural ORFs and a poly (A) tail [56, 57]. The ORF1a gene is located at the 5′UTR, encodes for polyprotein pp1a, which contains 10 nsps. The ORF1b gene, located next to ORF1a, encodes for polyprotein pp1ab which contains 16 nsps [55]. The pp1ab and pp1a protein undergoes autoproteolytic cleavage to form the viral replication complex. The 3′UTR contains the four structural genes and eight accessory genes. The accessory genes are distributed between the structural genes and their function is mostly unknown [55, 57]. The SARS-CoV-2 is a non-segmented enveloped virus with a diameter of 50–200 nm [58]. Structurally, it has a double-layered lipid envelope, including Spike glycoprotein (S), Envelope protein (E), Membrane glycoprotein (M), and Nucleocapsid protein (N) [59, 60]. The viral genome having a RBD for the interaction with host cell receptors is covered by the Spike glycoprotein [46]. The M glycoprotein is responsible for the assembly of viral particles has three domains, the cytoplasmic domain, the transmembrane domain, and the N hydrophilic domain [61]. The Envelope protein is reported to play role in pathogenesis as it interacts with the tight junction related protein PALS1 [62]. The nucleocapsid protein packs the viral genome into a ribonucleoprotein complex [63]. The nucleocapsid, a phosphoprotein plays role in viral genome replication and the cell signaling pathway (Fig. 2).

## Replication of SARS-CoV-2

The positive sense RNA genome serves as a template for both replication and protein synthesis. The virus enters through membrane fusion and releases its positive sense RNA into the cytoplasm. The CoVs control the relative expression of their proteins through a conserved molecular mechanism, known as -1 programmed ribosomal frameshifting (-1 PRF) [64]. The SARS-CoV-2 -1PRF and SARS-CoV1-1PRF is nearly similar with a single nucleotide difference which does not impact the rate of -1 PRF in SARS-CoV-2 [65].

The two ORFs of the viral genome, ORF1a and ORF1b translate to non-structural proteins (nsps) in the cytoplasm. The ORF1a produces a polypeptide pp1a, which proteolytically cleaved to produce 10nsps while the -1PRF of SARS-CoV-2 allows continued translation till ORF1b [2, 65, 66] and yields a larger polypeptide (pp1ab) which gets cleaved into 16 nsps. The proteolytic cleavage of the polypeptides is carried out by the viral proteases 3CL^pro^ and M^pro^ [2, 67]. The functions of different nsps are listed in Table 1.

**Table 1:** The functions of non-structural proteins of SARS-CoV-2

S. no	Proteins	Functions	Refs
1.	nsp1	Interferes with the mRNA binding and suppresses hosts’ immune functions Anchors the replication complex to cellular membranes Degrades host’s mRNA by interacting with the human 40S ribosomal subunit	[132, 133]
2.	nsp2	Harbours mutations that make it more contagious Might play a role in modulation of host cell survival; also known as p65 homolog	[132, 134]
3.	nsp3	Papain-like protease 2 (PL2^pro^) involved in proteolytic cleavage	[135]
4.	nsp4	Responsible for the formation of the double membrane vesicle during replication Anchors the viral replication-transcription complex to the membranes of endoplasmic reticulum	[136, 137]
5.	nsp5	Proteases (3CL^pro^, M^pro^) involved in polypeptide cleaving	[138]
6.	nsp6	Prevents the expansion of autophagosome, Help in formation of double membrane vesicle; suppresses IFN-I signaling	[139, 140]
7.	nsp7	Forms a hexadecamer with nsp8 and acts as a primase in viral replication	[141]
8.	nsp8	Acts as a primase with nsp7	[142, 143]
9.	nsp9	Acts as ssRNA binding protein	[143, 144]
10.	nsp10	Plays role in the methylation of viral mRNA cap. Stimulates the nsp14 3′-5′ exoribonuclease and 2′-O-methyltransferase (NSP16) activities	[144]
11	nsp11	Unknown	
12	nsp12	Catalytic subunit of the RNA-dependent RNA polymerase; Catalyses the synthesis of viral RNA, using nsp7 and nsp8 as cofactors	[142, 145, 146]
13.	nsp13	Helicase and NTPase activity: hydrolyze the NTPs and unwind the duplex RNA and DNA with a 5′ single-stranded tail in a 5′ to 3′ direction A potent interferon antagonist	[147–149]
14.	nsp14	Guanine-N7 methyltransferase, a multienzyme complex Acts on both sides ssRNA and dsRNA in a 3′-> 5′ direction A potent interferon antagonist It plays role in genome replication, sub-genomic RNA synthesis and recombination	[132, 148, 150]
15.	nsp15	It is a nidoviral RNA uridylate‐specific endoribonuclease (NendoU); plays role in viral replication and transcription A potent interferon antagonist	[148, 151, 152]
16.	nsp16	Acts as 2′-*O*-methyltransferase that mediates mRNA cap 2′-*O*-ribose methylation to the 5′-cap structure of viral mRNAs	[148, 153]

**Fig. 3:**
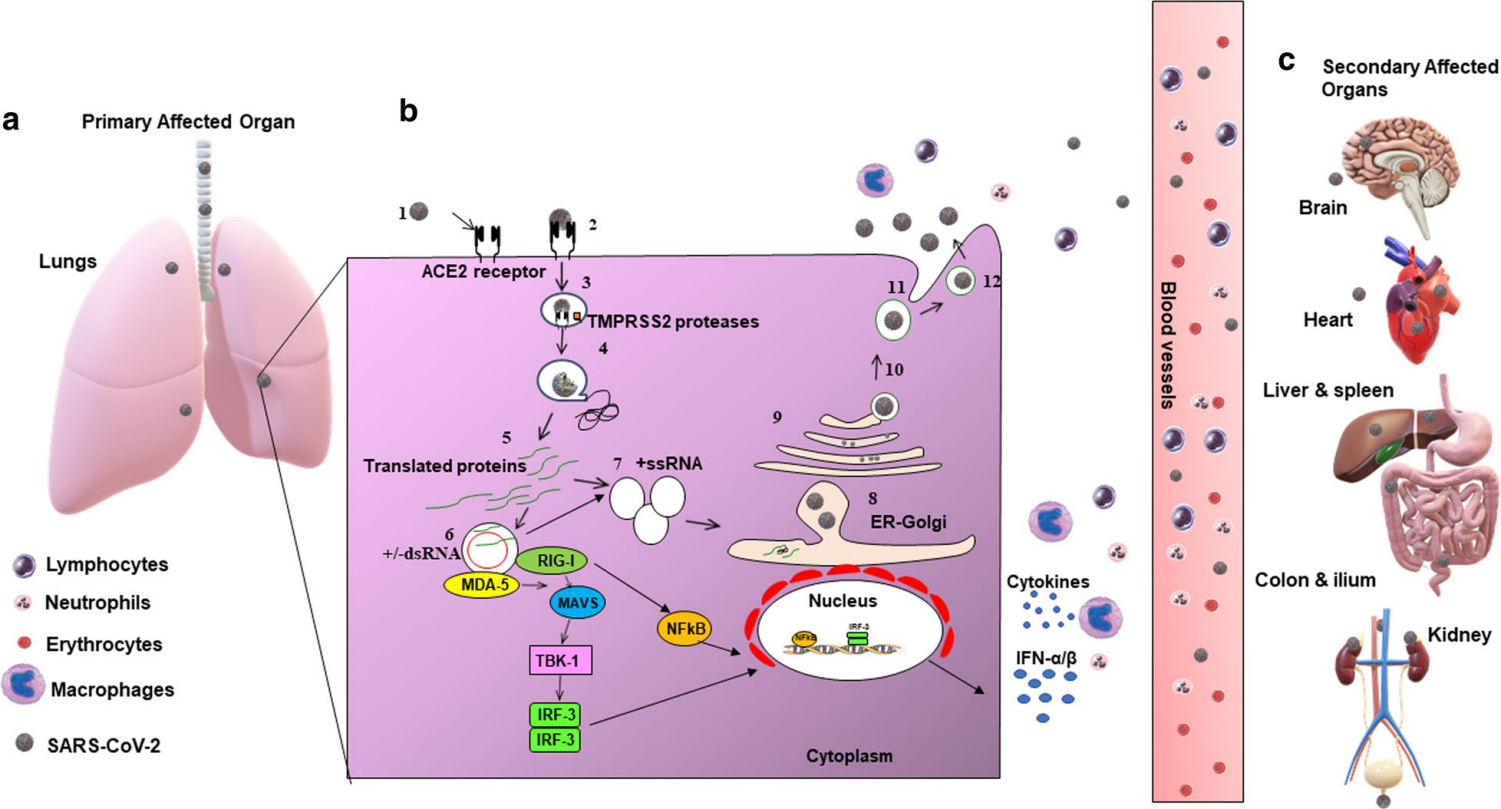
The SARS-CoV-2 replication and pathogenesis. **a** The SARS-CoV-2 infects upper and lower respiratory tract. **b** The virus replication from 1 to 12 has been described as, (1) The virus identifies the ACE-2 receptor. (2) The binding is initiated by the Spike (S) glycoprotein of SARS-CoV-2 by receptor binding domain (RBD) to the ACE-2 receptor binding motif (RBM). (3, 4) The virus-receptor internalization occurs and a membrane fusion is carried out by S2 subunit of S glycoprotein followed by the uncoating of +ssRNA inside cytoplasm. (5–7) The +ssRNA directly translate into non-structural proteins for initiating the viral replication through RNA-dependent RNA polymerase (RdRp/nsp12) and forms -ssRNA which act as template for synthesizing various copies of +ssRNA. (8–10) The +ssRNA along with the structural protein are trafficked to ER-Golgi complex for assembly and maturation. (11–12) The vesicles-encapsulated virion fuses with the cell membrane and through exocytosis release outside the cell to infect nearby cell. The immune response: the cellular RNA receptors like, RIG-I and MDA-5 recognized the dsRNAs in cytoplasm, activating the pro-inflammatory response and antiviral response inside cells. The cytokines activate the macrophages and lymphocytes to kick start both cellular and humoral response. **c** The virus disseminates to the other part of body through blood affecting the brain, heart, liver spleen, large intestine, kidneys

The replication and transcription of the viral genome is mediated by the activity of RNA dependent RNA polymerase (RdRP/nsp12). The RdRP catalyzes the synthesis of viral RNA, with the assistance of nsp7 and nsp8 as cofactors [68]. The RNA viruses lack the proofreading capacity. Although, Smith et al. reported that, an exoribonuclease domain (ExoN) in non-structural protein 14 provides proofreading activity that protects the SARS-CoV1 from mutagenesis [69]. The ExoN deletion leads to reduced replicative fidelity [69]. The replication complex generates a full-length negative sense RNA intermediates from the viral genome, which serve as the template for the synthesis of positive sense genomic RNAs (gRNA) and the sub-genomic RNA (sgRNA). The nucleocapsid protein encapsulates the gRNA, the S, M and E proteins in the endoplasmic reticulum-Golgi intermediate compartment. The assembly of mature virion occurs inside the Golgi and the virion containing vesicles fuse with plasma membrane and release the virus by exocytosis (Fig. 3b) [2, 66]. The SARS-CoV-2 expresses nine sgRNAs (S, 3a, E, M, 6, 7a, 7b, 8, and N) which form the structural and accessory proteins. These sgRNAs are produced by the canonical Transcription Regulatory Sequence (TRS) mediated mechanism for discontinuous transcription [57, 70].

## Host viral interactions during COVID-19 infection

### SARS-CoV-2 receptors

The CoVs enter inside cell using various cell surface receptors. The ACE2 are used by SARS-CoV-1 and HCoV-NL63, whereas MERS uses dipeptidyl peptidase-4 (DPP4) and HCoV-229E uses aminopeptidase N (APN) [71–74]. All CoVs employ S glycoprotein for their internalization. The S glycoprotein has two subunits, S1 and S2. The S1 subunit comprises of the receptor binding domain (RBD), which binds with the receptor binding motif (RBM) of cell surface receptor, while the S2 subunit, mediates the fusion of host-virus cell membrane [75–77]. The S^B^ domain of S1 subunit is a RBD of SARS-CoV-1, which mostly binds with the ACE2 receptor for their internalization [72, 74, 78]. The S protein is cleaved by host proteases at S’2 site (located on S2 sub-unit) to make necessary conformational changes for membrane fusion [76, 79]. The type II transmembrane serine protease (TMPRSS2) is the main host protease that mediates S protein activation and initial viral entry in primary target cells [47, 80, 81]. The camostat mesylate, an inhibitor of host serine protease TMPRSS2 blocks the entry of CoVs in the cell. This indicates that the role of TMPRSS2 is important in priming the S glycoproteins for successfully coordinating the events of SARS-CoV2 [81]. The furin is another host protease, which has been suggested to play an important role in the SARS-CoV-2 pathogenesis [47]. The structural analysis of SARS-CoV-1 Spike glycoprotein and SARS-CoV-2 Spike glycoprotein reveals 76% similarity in the amino-acid sequences [47, 56]. Moreover, both SARS-CoV-1 and SARS-CoV-2 shared 8 conserved binding positions and six semi-conserved positions in their S^B^ domain [47, 82]. Therefore, the binding efficiency of Spike glycoproteins of both SARS-CoV-1 and SARS-CoV-2 are similar. However, the conservation and semi-substitution in SARS-CoV-2, spike glycoprotein had somehow made the SARS-CoV-2 more adaptable to ACE2 receptor, thereby increasing the transmissibility in humans [47].

### Immune response during SARS-COV-2 infection

The SARS-CoV-2 disseminates to various organs after infecting the upper and lower respiratory tract. The ACE2 receptor are highly expressed on the type II alveolar epithelial cells, airway epithelium, lung parenchyma, esophagus epithelial cells, enterocytes from ilium and colon, myocardial cells, cholangiocytes in liver and the proximal tubule cells in kidney [12, 13]. The SARS-CoV-2 RNA is recognized by endosomal TLR7, TLR8 or by RNA sensors like, MDA5 and RIG-1. Upon activation, the signaling cascade including, NF-kB transcription, AP-1 induces the gene responsible for the production of pro-inflammatory cytokines like, TNF-α, TGF-β, IL-1β, IL-6, IL-8, IL-12, IL-18 and chemokines like, CCL2, CCL3, CCL5, CXCL8, CXCL9, CXCL10. Therefore, a cytokine storm is generated which either directly, indirectly or synergistically damages the organs or may lead to acute respiratory distress syndrome (ARDS) or multiple organ failure in COVID-19 patients [28, 58, 83, 84]. Li et al. 2020, reported that nsp9 and nsp10 of SARS-CoV-2 interacts with NKRF to mediate the IL-6/IL-8 signaling leading to the uncontrolled activation and infiltration of neutrophils from the periphery [85]. The COVID-19 patients have been reported with decrease in the numbers of CD4^+^ and CD8^+^ cells in peripheral blood, which may lead to secondary bacterial infection and increases the disease severity. MERS-CoV can directly infect the T-lymphocytes, promote their apoptosis and causes lymphopenia [86]. Wang et al., 2020 reported, SARS-CoV-2 can directly infect T-lymphocytes by S glycoprotein through membrane fusion [87]. In addition, lack of T-cells may not activate the antibody-producing B-lymphocytes, which affects the production of immunoglobulins in COVID-19 patients [58, 88]. Moreover, the ADE towards similar epitopes against S or N glycoproteins of SARS-CoV-1 may lead to ARDS or multiple organ failure [89].

The heterogeneous roles of CD4^+^ T and CD8^+^ T have been reported in both, SARS-CoV-2 and other respiratory-related viral infections [90, 91]. There are reports which have highlighted the importance of CD8^+^ T cells in COVID-19 patients. The patients with mild COVID-19 symptoms or COVID-19 recovered patients have SARS-CoV-2 specific CD8^+^ T cells response in 70% of the cases. It has also been shown that CD8^+^ T cells were specific to viral internal proteins and can be considered for vaccine development [92, 93]. The antiviral response mediated by IRF3 and IRF7 release the IFN-α/β and IFN-γ which have a protective role in suppressing the viral spread at the later stages of infection, as seen in SARS-CoV-1 and MERS. The virus circumvents the host immune response by suppressing the type I interferon as shown in mouse model of SARS-CoV-1 and MERS [94, 95]. Lokugamage et al. 2020 reported that SARS-CoV-2 is more sensitive to type-I IFN treatment than the SARS and MERS-CoVs. In addition, they reported that the mutation in ORF3b and truncation of ORF6 have rendered SARS-CoV-2 more susceptible to type-I IFN treatment (Fig. 3) [85, 94, 96].

## Clinical presentation, pathophysiology and diagnosis of COVID-19 patients

The COVID-19 clinical presentations are similar to that of SARS-CoV1 and MERS, which are mild and self-limiting in 80% of the cases. Only 20% of the cases aggravate to secondary complications of ARDS or multiple organ failure [97, 98]. The virus affects people differently depending upon the genetic pre-disposition, immune status and diseases associated with respiratory system [99, 100]. The people > 60 years of age are at higher risk of exacerbating the disease [58, 83, 101].

The incubation period for SARS-COV-2 has been estimated up to 14 days, which is longer than SARS-CoV-1 and MERS [102, 103]. The longer incubation period supports the asymptomatic and subclinical infection rate [14]. The common symptoms include fever, dry cough, fatigue, myalgia, dyspnea, runny nose, nausea, joint pain and gastrointestinal symptom [104]. In addition, patients with co-morbidities like, diabetes, hypertension [28, 83, 84], acute kidney disease, cardiac problem, cerebrovascular disease or liver dysfunction may be more susceptible to infection [97, 105].

The severity of COVID19 directly co-relates to lymphopenia, eosinopenia and hypercytokinemia, similar to SARS-CoV-1 and MERS [83, 84, 97, 106]. The serological reports of COVID-19 patients show a sharp increase in their C reactive proteins, lactate dehydrogenase (LDH), erythrocyte sedimentation rate (ESR), creatinine kinase, alanine aminotransferase (ALT), aspartate transaminase (AST), D-dimer and low serum albumin [13, 105, 107] indicating sepsis which may lead to multiorgan failure during the later stages of infection. The higher levels of pro-inflammatory cytokines like, IL2, IL7, IL10, granulocyte-colony stimulating factor (GCSF), interferon-gamma protein-10 (IP10), monocyte chemoattractant protein-1 (MCP1), macrophage inflammatory protein-1α (MIP1A), and TNFα may contribute to “cytokine storm” similar to SARS-CoV1 and MERS [83, 107–110]. The CT-scans and X-Ray reports of COVID-19 patients revealed the opacities and bilateral diffuse alveolar damage followed by cellular exudates, pleurisy, pericarditis, lung consolidation and pulmonary edema [13, 109]. The autopsy reports revealed the atypical enlarged pneumocytes, interstitial mononuclear infiltration with significant cytopathic effects and the presence of lymphocytes in the affected area and thick alveolar wall [83]. The other deformities include degeneration of neurons, atrophy of spleen, infiltration of immune cells in liver, hyaline thrombus in blood vessels leads to cardiac arrest and hemorrhage in kidney are observed in severely affected COVID-19 patients [13, 109–111]. The nasopharyngeal swab/oropharyngeal swab (upper respiratory tract), sputum, lavage or aspirate (lower respiratory tract) is used for diagnosis. In addition, blood, stool and urine sample are also used for need based diagnosis [112]. The diagnosis is carried by RT-qPCR or by high throughput sequencing of viral genome. The primers and probes against ORF1b and N genes are used for the detection of SARS-COV-2 from respiratory fluid [113]. The asymptomatic patients are identified by the presence of viral nucleic acid, and are responsible for human-to human transmission of SARS-COV-2 infection [101, 114–116]. Many molecular based diagnostics and immunoassays are used for the detection of SARS-COV-2 (Table 2).

**Table 2:** Diagnostic kits for COVID-19

S. No	Name	Company name	Regulatory/Authorization	Refs
*Molecular assays for COVID-19 diagnosis*				
1.	ANDiS® SARS-CoV-2 RT-qPCR Detection Kit	3D Medicine Science & Technology Co., Ltd	US FDA-EUA—CE-IVD	[154]
2.	Abbott RealTime SARS-CoV-2 EUA test	Abbott Molecular Inc	US FDA-EUA—CE-IVD	[155]
3.	Truenat™ Beta CoV (lab-based or near-POC)	Molbio Diagnostics Pvt Ltd	India DCGI	[156]
4.	QIAstat-Dx Respiratory Panel 2019-nCoV	QIAGEN GmbH	US FDA-EUA—CE-IVD	[157]
5.	cobas® SARS-CoV-2 (for use on the cobas® 6800/8800 Systems)	Roche Molecular Diagnostics	US FDA-EUA—WHO EUL	[158]
6.	Senteligo Covid-19 qRT PCR Detection Kit	Sente Biolab	CE-IVD	[159]
7.	VereCoV™ Detection Kit	Veredus Laboratories Pte Ltd	Singapore HSA—CE-IVD	[160]
8.	VISION COVID19 Easyprep Test Kit	Vision Biotechnology Research & Development	IFA ISO 9001: 2015	[161]
9.	ePlex® SARS-CoV-2 Test	GenMark Diagnostics	US-FDA EUA	[162]
*Immunoassays for COVID-19 diagnosis*				
10.	Accu-Tell COVID-19 IgG/IgM Rapid Test Cassette Specimen: Whole blood/Serum/Plasma	AccuBioTech Co. Ltd	CE-IVD	[163]
11.	SARS-CoV-2 IgM/IgG antibody test kit (Colloidal Gold Method)	BIOHIT HealthCare (Hefei) Co., Ltd	CE-IVD	[164]
12.	COVID-19 IgM-IgG Dual Antibody Rapid Test	BioMedomics, Inc	CE-IVD	[165]
13.	Cellex qSARS-COV-2 IgG/IgM Rapid Test Specimen: Whole blood/Serum/Plasma	Cellex Inc	US FDA-EUA—CE-IVD	[166]
14.	Human Anti-SARS-CoV-2 (Covid-19) IgG/IgM Rapid Test	KRISHGEN BioSystems	CE-IVD	[167]
15.	SARS-CoV-2 IgM/IgG Ab Rapid Test Specimen: WB/S/P	Sure Bio-Tech (USA) Co., Ltd	CE-IVD	[168]

## Vaccines and therapeutics

Scientists across the world are aiming to instigate therapeutics and vaccine against SARS CoV-2. The vaccine should be useful for all age groups and people with various co-morbidities. According to WHO report; there are 48 vaccine candidates under the advance stages of the clinical trials (Table 3). Among them 11 are currently in clinical trial Phase-III. The vaccine produced by BioNTech/Pfizer BNT162b2, is in the late stage of clinical trial. The BNT162b2is an mRNA-based vaccine; which exploits for RBD of SARS CoV-2 spike protein for eliciting immune response. This mRNA vaccine is lipid encapsulated for its effective delivery into the cells [117]. Another RNA based vaccine mRNA1273 is in clinical phase-III trial. This vaccine has been developed by Moderna Incorporation. The mRNA1273 vaccines lipid-nanoparticle encapsulated mRNA encoding for the SARS-CoV-2 spike glycoprotein [118]. The other potential vaccine is of University of Oxford with AstraZeneca (AZD1222 vaccine) is also currently at the final stages of the clinical trials. The AZD1222 vaccine has been developed by using the Chimpanzee adenovirus viral vector, ChAdOxo-1s, which encodes for SARS-CoV-2 spike protein [119]. Another viral vector-based vaccine Sputnik V has been developed by Gamaleya Research Institute in Russia. The vaccine contains 2 viral vectors recombinant adenovirus type 26 (rAd26) and recombinant adenovirus type 5 (rAd5) encoding the full length S glycoprotein of SARS-CoV-2 [120].

**Table 3:** List of candidate vaccines against COVID-19. This table has been taken with permission from the WHO website (Draft landscape of COVID19 candidate vaccine) with slight modifications [169]

S. no	Vaccine developer	Platform	Type of candidate vaccine	Current status
1.	Sinovac	Inactivated	Inactivated	Phase-3
2.	Wuhan Institute of Biological Products/Sinopharm	Inactivated	Inactivated	Phase-3
3.	Beijing Institute of Biological Products/Sinopharm	Inactivated	Inactivated	Phase-3
4.	Bharat Biotech	Inactivated	Whole virion inactivated	Phase-3
5.	University of Oxford/AstraZeneca	Non-replicating viral vector	ChAdOx1-S	Phase-3
6.	CanSino Biological Incorporation/Beijing Institute of Biotechnology	Non-replicating viral vector	Adenovirus Type 5 Vector	Phase-3
7.	Gamaleya Research Institute	Non-replicating viral vector	Adeno-based (rAd26-S + Ad5-S)	Phase-3
8.	Janssen Pharmaceutical Companies	Non-replicating viral vector	Ad26COVS1	Phase-3
9.	Novavax	Protein subunit	Full length recombinant SARS COV-2 glycoprotein nanoparticle Vaccine adjuvanted wih Matrix M	Phase-3
10.	Moderna/NIAID	RNA	LNP-encapsulated mRNA	Phase-3
11.	BioNTech/Fosum Pharma/Pfizer	RNA	3 LNPs mRNA	Phase-3
12.	Beijing Wantai Biological Pharmacy/Xiamen University	Replicating viral vector	Intranasal flu based RBD	Phase-2
13.	Anhui ZhifeiLongcom Biopharmaceutical/Institute of Microbiology, Chinese Academy of Sciences	Protein subunit	Adjuvanted recombinant protein (RBD-dimer)	Phase-2
14.	Curevac	RNA	mRNA	Phase-2
15.	Institute of Medical Biology/Chinese Academy of Medical Sciences	Inactivated	Inactivated	Phase-1/2
16.	Research Institute for Biological Safety Program, Rep of Kazakhstan	Inactivated	Inactivated	Phase-1/2
17.	Beijing Minhai Biotechnology Co., Ltd	Inactivated	Inactivated	Phase-1/2
18.	Inovio Pharmaceuticals/International Vaccine Institute	DNA	DNA plasmid vaccine with electroporation	Phase-1/2
19.	Osaka University/ AnGes/ Takara Bio	DNA	DNA plasmid vaccine with adjuvant	Phase-1/2
20.	Cadila Healthcare Limited	DNA	DNA plasmid vaccine	Phase-1/2
21.	Genexine Consortium	DNA	DNA Vaccine (GX-19)	Phase-1/2
22.	Kentucky Bioprocessing Inc	Protein subunt	RBD-based	Phase-1/2
23.	Sanofi Pasteur/ GSK	Protein subunit	S-protein Baculovirus production	Phase-1/2
24.	Biological E Ltd	Protein subunit	Adjuvanted Protein subunit (RBD)	Phase-1/2
25.	Israel Institute for Biological Research	Replicating viral vector	VSV-S	Phase-1/2
26.	Arcturus/ Duke-NUS	RNA	mRNA	Phase-1/2
27.	Spy Biotech/Serum Institute of India	VLP	RBD-HBSAg VLPs	Phase-1/2
28.	Symvivo	DNA	bacTRL-spike	Phase-1
29.	Immunity Bio, Inc. &Nanktwest Inc	Non replicating viral vector	hAd5 S + N 2nd Generation Human Adenovirus Type 5 Vector (hAd5) Spike (S) + Nucleocapsid (N)	Phase-1
30.	Reithera/LEUKOCARE/Univercells	Non replicating viral vector	Replication defective Simian Adenovirus encoding S protein	Phase-1
31.	Cansino Biological Inc	Non replicating viral vector	Ad5-nCoV	Phase-1
32.	Vaxart	Non replicating viral vector	Ad5 Adujvant oral vaccine platform	Phase-1
33.	Ludwig-Maximillians-University of Munich	Non replicating viral vector	MVA SARS-2-S	Phase-1
34.	Clover Biopharamaceuticals Inc./GSK/ Dynavax	Protein subunit	Native like trimeric subunit spike protein vaccine	Phase-1
35.	Vaxine Pty Ltd/ Medytox	Protein subunit	Recombinant spike protein with Advax adjuvant	Phase-1
36.	University of Queensland/CSL/Seqirus	Protein subunit	Molecular clamp stabilized spike protein with MF59 adjuvant	Phase-1
37.	Medigen Vaccine Biologics Corporation/NIAID/Dynavax	Protein subunit	S-2p protein and CpG 1018	Phase-1
38.	Instituto Finlay de Vacunas, Cuba	Protein subunit	rRBD produced in CHO cell chemically conjugate to tetanus toxoid	Phase-1
39.	Instituto Finlay de Vacunas, Cuba	Protein subunit	RBD + Adjuvant	Phase-1
40.	FBRI SRC VB VECTOR, Rospotrebnadzor, Koltsovo	Protein subunit	Peptide	Phase-1
41.	West China Hospital, Sichuan University	Protein subunit	RBD Baculovirus production expressed in Sf9 cells	Phase-1
42.	University Tuebingen	Protein subunit	SARS-CoV-2 HLA-DR peptides	Phase-1
43.	COVAXX/United Biomedical Inc. Asia	Protein subunit	Multitope peptide based S1 RBD protein vaccine	Phase-1
44.	Merck Sharp & Dohme/IAVI	Replicating viral vector	Replication competent VSV delivering SARS-CoV-2 spike	Phase-1
45.	Institute Pasteur/Themis/Univ. of Pittsburg CVR/Merck Sharp & Dhome	Replicating viral vector	Measles vector based	Phase-1
46.	Imperial College London	RNA	LNP nCoVsaRNA	Phase-1
47.	People’s Liberation Army, Academy of Military Sciences/Walvax Biotech	RNA	mRNA	Phase-1
48.	Medicago Inc	VLP	Plant derived VLP adjuvanted with GSK or Dynavax adjs	Phase-1

The different classes of therapeutics that can be used for the treatment of COVID-19 patients include, protease inhibitors, nucleoside analogue, neutralizing-monoclonal antibody, immune modulator, RNA polymerase inhibitor, interferon alpha, endonuclease Inhibitor, fusion inhibitor, convalescence plasma therapy and Immunosuppressant [121].

The antimalarial drug, Ivermectin has been tried in combination with Remedesivir for the COVID-19 treatment. Ivermectin has been reported to inhibit the nuclear translocation of viral protein and prevents the inhibition of antiviral response [122, 123].

Remedesivir (GS-5734) is an adenosine analog, which suppresses the viral replication by interfering in the activities of RdRp. Remedesivir is also used against MERS, SARS-CoV1 [124–126]. Bamlanivimab (LY-CoV555), the monoclonal antibody produced by Eli Lilly & Company has been granted an emergency approval by FDA for COVID-19 treatment. It is a recombinant human monoclonal neutralizing antibody IgG1 produced against SARS-CoV-2 spike protein [127]. The Hydroxychloroquine (HCQ) a well-known antimalarial drug has been under trial for COVID 19 treatment but it was not found to have any beneficial effect for the COVID19 patients. HCQ was reported to inhibit the viral infection by glycosylating the ACE2 receptor of SARS-CoV-1 and increases the endosomal pH, rendering the membrane fusion [124, 128]. Favipiravir has also been under trial for the treatment of COVID 19 patients as it was previously tested against Ebola and Influenza virus. Favipiravir inhibits the RNA polymerases and halts the viral replication [129]. The convalescent plasma therapy has been used for treating the critical COVID-19 patients during the early phases of this outbreak. Previously it was used against SARS-CoV-1, Ebola and H1N1 but the use of convalescent plasma therapy against SARS-CoV2 has been quite debatable issue [130]. Another type of therapeutic, tocilizumab has also been used against SARS-CoV2. Tocilizumab has been used for the treatment of severe rheumatoid arthritis, systemic juvenile idiopathic arthritis and giant cell arteritis. Tocilizumab, an immunosuppressant binds to the IL-6 receptor and hinders the inflammatory responses [131]. In addition, there are many other therapeutic trials for COVID-19 ongoing and some of them with positive responses have been listed in Table 4.

**Table 4:** List of therapeutic candidates against COVID-19

S. no	Drug name	Clinical trial	Trial	Treatment	References
1.	Pacritinib	Phase 3	NCT04404361	Kinase inhibitor	[170]
2.	Enoxaparin	Phase 3	NCT04401293	Antithrombotic	[171]
3.	Remdesivir + Baricitinib	Phase 3	NCT04401579	Antiviral	[172]
4.	Remdesivir	Phase 3	NCT04401579	Antiviral	[172]
5.	Hydroxychloroquine	Phase 3	NCT04410562	Antimalarial	[173]
6.	Favipiravir + Hydroxychloroquine	Phase 3	NCT04411433	Antiviral	[174, 175]
7.	ASC09 + Oseltamivir	Phase 3	NCT04261270	Antiviral	[176]
8.	ASC09 + Ritonavir	NA	NCT04261907	Antiviral	[176]
9.	Tocilizumab (IL-6)	Phase 3	NCT04412772	Monoclonal antibodies	[177]
10.	Anakinra	Phase 3	NCT04412291	Anti-inflammatory	[178]
11.	Ivermectin	Completed	NCT04422561	Antiparasitic	[179]
12.	Budesonide dry powder inhaler	Phase 2	NCT04416399	Corticosteroid	[180]
13.	LY3819253	Phase 3	NCT04427501	Corticosteroid	[181]
14.	Atazanavir and dexamethasone	Phase 3	NCT04452565	Corticosteroid and antiviral	[182]
15.	Colchicine	Phase 2	NCT04326790	Anti-inflammatory	[183]
16.	Corticosteroid	Phase 3	NCT04381936	Corticosteroid	[184]
17.	Azithromycin	Phase 3	NCT04381936	Antibacterial	[184]
18.	Convalescent plasma	Phase 3	NCT04425915	Convalescent plasma	[130]
19.	NA-831 and dexamethasone	Phase 3	NCT04452565	Corticosteroid	[182]
20.	Camostat Mesilate	Phase 2	NCT04470544	Protease inhibitor	[81]

## Data Availability

Not applicable.

## Abbreviations

CoVs: Coronavirus
SARS-CoV-1: Severe acute respiratory syndrome coronavirus 1
SARS-CoV-2: Severe acute respiratory syndrome coronavirus 2
MERS-CoV: Middle East respiratory syndrome coronavirus
ICTV: International Committee on Taxonomy of Viruses
PHEIC: Public Health Emergency of International Concern
ACE2: Angiotensin converting enzyme-2
DPP4: Dipeptidyl peptidase-4
APN: Aminopeptidase N
HCoV-229E: Human Coronavirus-229E
COPD: Chronic obstructive pulmonary diseases
CVD: Cardiovascular disease
RBD: Receptor Binding Domain
RBM: Receptor Binding Motif
nsps: Non-structural proteins
-1 PRF: -1 Programmed ribosomal frameshifting
ExoN: Exoribonuclease domain
gRNA: Genomic RNA
sgRNA: Sub-genomic RNA
TRS: Transcription Regulatory Sequence
TMPRSS2: Type II transmembrane serine protease
MDA-5: Melanoma Differentiation‐Associated gene 5
RIG-1: Retinoid‐Inducible Gene-1
ARDS: Acute respiratory distress syndrome
ADE: Antibody-dependent-enhancement
IRF3 and 7: Interferon response factor 3, and 7
LDH: Lactate dehydrogenase
ESR: Erythrocyte sedimentation rate
ALT: Alanine aminotransferase
AST: Aspartate transaminase
GCSF: Granulocyte-colony stimulating factor
IP-10: Interferon-gamma protein-10
MCP-1: Monocyte chemoattractant protein-1
MIP1A: Macrophage inflammatory protein-1α
qRT-PCR: Quantitative real time polymerase chain reaction
POCT: Point-of-care-testing
RdRp: RNA dependent RNA polymerase
HCQ: Hydroxy-chloroquine sulphate
